# Dipeptidyl Peptidase IV Inhibitory Peptides from Chickpea Proteins (*Cicer arietinum* L.): Pharmacokinetics, Molecular Interactions, and Multi-Bioactivities

**DOI:** 10.3390/ph16081109

**Published:** 2023-08-04

**Authors:** José Antonio Mora-Melgem, Jesús Gilberto Arámburo-Gálvez, Feliznando Isidro Cárdenas-Torres, Jhonatan Gonzalez-Santamaria, Giovanni Isaí Ramírez-Torres, Aldo Alejandro Arvizu-Flores, Oscar Gerardo Figueroa-Salcido, Noé Ontiveros

**Affiliations:** 1 Nutrition Sciences Postgraduate Program, Faculty of Nutrition Sciences, Autonomous University of Sinaloa, Culiacan 80010, Mexico; joseantoniomoramelgem@gmail.com (J.A.M.-M.); gilberto.aramburo@uas.edu.mx (J.G.A.-G.); feliznando@uas.edu.mx (F.I.C.-T.); jgonzalez@utp.edu.co (J.G.-S.); giovanni.ramirez@uas.edu.mx (G.I.R.-T.); 2 Faculty of Health and Sports Sciences, University Foundation of the Andean Area, Pereira 66001, Colombia; 3 Faculty of Physical Education and Sports, Autonomous University of Sinaloa, Culiacan 80013, Mexico; 4 Postgraduate Program in Health Sciences, Faculty of Biological and Health Sciences, University of Sonora, Hermosillo 83000, Mexico; aldo.arvizu@unison.mx; 5 Integral Postgraduate Program in Biotechnology, Faculty of Chemical and Biological Sciences, Autonomous University of Sinaloa, Ciudad Universitaria, Culiacan 80010, Mexico; 6 Clinical and Research Laboratory (LACIUS, CN), Department of Chemical, Biological, and Agricultural Sciences (DCQBA), Faculty of Biological and Health Sciences, University of Sonora, Navojoa 85880, Mexico

**Keywords:** chickpea, bioactive peptides, DPP-IV inhibitors, in silico, molecular docking, ADMET

## Abstract

Chickpea (*Cicer arietinum* L.) peptides can inhibit dipeptidyl peptidase IV (DPP-IV), an important type 2 diabetes mellitus therapeutic target. The molecular interactions between the inhibitory peptides and the active site of DPP-IV have not been thoroughly examined, nor have their pharmacokinetic properties. Therefore, the predictions of legumin- and provicilin-derived DPP-IV inhibitory peptides, their molecular interactions with the active site of DPP-IV, and their pharmacokinetic properties were carried out. Ninety-two unique DPP-IV inhibitory peptides were identified. Papain and trypsin were the enzymes with the highest A_E_ (0.0927) and lowest B_E_ (6.8625 × 10^−7^) values, respectively. Peptide binding energy values ranged from −5.2 to −7.9 kcal/mol. HIS-PHE was the most potent DPP-IV inhibitory peptide and interacts with residues of the active sites S1 (TYR662) and S2 (GLU205/ARG125 (hydrogen bonds: <3.0 Å)), S2 (GLU205/GLU206 (electrostatic interactions: <3.0 Å)), and S2′ pocket (PHE357 (hydrophobic interaction: 4.36 Å)). Most peptides showed optimal absorption (76.09%), bioavailability (89.13%), and were non-toxic (97.8%) stable for gastrointestinal digestion (73.9%). Some peptides (60.86%) could also inhibit ACE-I. Chickpea is a source of non-toxic and bioavailable DPP-IV-inhibitory peptides with dual bioactivity. Studies addressing the potential of chickpea peptides as therapeutic or adjunct agents for treating type 2 diabetes are warranted.

## 1. Introduction

Insufficient production of and resistance to insulin are characteristics of type 2 diabetes mellitus (DM2) [1]. There are therapeutic agents available for treating approximately 537 million adults with DM2 (around 10.5% of the adult population aged 20 to 79 years) [2], but their long-term usage could develop adverse effects such as headaches, urinary tract infections, arthralgia, hypersensitivity to gliptins, and pancreatitis [3,4]. Dipeptidyl peptidase IV (DPP-IV) is a ubiquitous proteolytic enzyme involved in the degradation of incretin hormones such as glucagon-like peptide 1 (GLP-1) and glucose-dependent insulinotropic polypeptide (GIP) [5]. These hormones assist in diverse biological processes, such as reducing postprandial plasma glucose levels, enhancing insulin synthesis, preserving pancreatic beta-cell function, facilitating peripheral glucose uptake and elimination, moderating gastric emptying rate, bolstering glucose metabolism, and promoting satiety [6]. Therefore, since DPP-IV inhibition increases the incretin system, DPP-IV inhibitors have been recognized as crucial therapeutic agents for managing DM2.

Because of their anticipated low or null toxicity [7] and potential antihypertensive, antioxidant, antitumor, and antidiabetic effects [8,9], interest in food-derived bioactive peptides (BPs) has increased. In this context, the identification of BPs from different sources can potentiate the production of ingredients for functional food development or lay the groundwork for peptide-based therapies. Bioinformatic tools have facilitated the identification of BPs, saving time working on the laboratory bench and, consequently, saving human and economic resources. Particularly, chickpea protein hydrolysates can inhibit DPP-IV in vitro [10,11], highlighting the potential use of chickpea peptides for treating DM2. However, the prediction of chickpea antidiabetic peptides and their pharmacokinetics remain largely unexplored, as do the molecular interactions between these peptides and DPP-IV. Therefore, to expand our knowledge about the predicted DPP-IV inhibitory peptides, in silico enzymatic hydrolyses of chickpea legumin and provicilin, as well as ADMET pharmacokinetic and molecular docking analyses of the identified antidiabetic peptides, were carried out in the present study.

## 2. Results and Discussion

### 2.1. Profile of Peptides Released after Enzymatic Hydrolysis

Enzymatic hydrolysis of dietary sources can produce DPP-IV inhibitory peptides [12]. In fact, antidiabetic peptides can be produced through alcalase or papain digestion [13]. Additionally, bromelain and ficin are inexpensive proteolytic enzymes with high specificity for hydrolysis, and can be used to produce bioactive peptides [14,15]. On the one hand, ficin is an enzyme extract composed of sulfhydryl proteases obtained from the latex of *Ficus carica*. This enzyme hydrolyzes diverse peptide bonds, but those following an aromatic residue are preferentially hydrolyzed by ficin [14]. The bioactivity of ficin hydrolysates and peptides has been evaluated in different in vitro models. For instance, ficin hydrolysates have shown the potential to inhibit the proliferation of breast cancer cell lines, such as MCF-7 and MDA-MB-231, and peptides released after the hydrolysis of proteins from different food matrices with ficin have shown antimicrobial, antioxidant, antihypertensive, and antidiabetic potential [16]. Supported by these and other findings, ficin is one of the most widely utilized vegetable enzymes for producing bioactive hydrolysates or peptides [14]. On the other hand, bromelain is a protease found in generous quantities in pineapple, and many reagent suppliers offer this enzyme for sale [15]. Both angiotensin-converting enzyme I (ACE-I) and DPP-IV inhibitory peptides can be released after the hydrolysis of proteins with bromelain. Abadía-García et al. [17] reported that whey protein hydrolysates obtained after hydrolysis with bromelain can inhibit ACE-I in vitro. Although the authors stated that ultrasound treatment of whey proteins before hydrolysis improves the ACE-I inhibitory activity of the peptides released, inhibitory peptides are released without the need for such a treatment [17]. Others reported that DPP-IV inhibitory peptides could be released after the hydrolysis of chickpea proteins with bromelain, but also stated that peptides released after pepsin and pancreatin digestion could have greater potential for inhibiting DPP-IV than those released after hydrolysis with bromelain [11]. Therefore, the present study included in silico alcalase, papain, bromelain, and ficin hydrolysis. Pepsin, trypsin, and chymotrypsin hydrolysis were also carried out, and to mimic gastrointestinal digestion, sequential hydrolysis with these three enzymes was performed. The likelihood of discovering DPP-IV inhibitory peptides increases when proteins are broken down by the aforementioned enzymes because a wide variety of peptides can be produced.

In this study, 290 and 275 DPP-IV inhibitory peptides from chickpea encrypted in the whole legumin and provicilin protein sequences were identified using the BIOPEP-UWM platform, respectively. After in silico enzymatic hydrolysis, a total of 191 legumin and 190 provicilin DPP-IV inhibitory peptides were released (Supplementary Materials Tables S1–S8). Among the 381 peptides identified, 376 were dipeptides and 5 were tripeptides. However, only 92 of them were unique. Notably, peptides of up to 10 amino acids have been reported to have low IC_50_ values and low binding energy in docking analyses [17,18]. These peptides were released after proteolysis of donkey blood, proteins from Brassica napus seeds, and chickpea proteins. These substrates were hydrolyzed with protease K, alcalase and trypsin, and pepsin and pancreatin, respectively [17,18,19]. It should be noted that the DPP-IV inhibitory peptides were identified using liquid chromatography with MS/MS detection, and in silico analyses were further carried out. Notably, a thirteen-amino acid peptide with a high energy of affinity with DDP-IV was released after the hydrolysis of chickpea protein with bromelain [19]. However, tetrapeptides or larger peptides could be hydrolyzed by digestive proteases, potentially losing their bioactivity in an in vivo model [20]. Contrarily, dipeptides and tripeptides are generally more resistant to gastrointestinal digestion than larger peptides, allowing for their bioavailability [12]. Certainly, ingested peptides are exposed to a variety of gastric and pancreatic proteases, as well as brush border peptidases such as pepsin, trypsin, chymotrypsin, and carboxypeptidases, among others. The peptide transport from the intestine to systemic circulation can occur through different mechanisms, such as the paracellular and transcellular routes and through peptide transporter 1 (a transmembrane protein found in the brush border) [21]. The paracellular route has a preference for di- and tripeptides with neutral or positive charges. Due to the lipidic composition of cell membranes, the transcellular route preferentially permeates hydrophobic peptides with estimated molecular weights of up to 700 Da. Regarding peptide transporter 1, this transport system is independent of the physicochemical properties of the di- and tripeptides that can be transported into the enterocyte [22]. Previous research highlights that peptide size could be more relevant than their net charge, hydrophobicity, or lipophilicity for becoming bioavailable, and that two-to-five amino acid peptides are most likely to reach the bloodstream [22]. In fact, in vitro studies show that DPP-IV inhibitory di- and tripeptides have lower binding energy in molecular docking analysis, and lower IC_50_ values than larger peptides, indicating their high potential to inhibit DPP-IV [5]. It should be noted that peptides that resist gastrointestinal digestion and become bioavailable are exposed to plasma peptidases, potentially limiting their half-life.

The frequency and potential of chickpea DPP-IV inhibitory peptides identified are shown in Table 1. Both chickpea proteins assessed are sources of DPP-IV inhibitory peptides and have similar A values, but legumin has sequences with higher DPP-IV inhibitory potential than provilicin (Table 1). After the enzymatic hydrolysis of provicilin, trypsin digestion releases peptides with the highest DPP-IV inhibitory potential. However, a higher quantity of DPP-IV inhibitory peptides is more likely to occur after bromelain or papain hydrolysis of provicilin (Table 1). Regarding legumin, the most abundant protein in chickpea seed [23], hydrolysis with papain or bromelain was more efficient in producing DPP-IV inhibitory peptides than hydrolysis performed with other enzymes evaluated in the present study (Table 1). The hydrolysis performed with alcalase or pepsin released peptides with the highest DPP-IV inhibitory potential (Table 1). Notably, pepsin hydrolyze peptide bonds with aromatic amino acids in the C-terminal region [24] and DPP-IV inhibitory peptides frequently have a hydrophobic or aromatic amino acid [25]. Overall, the results show that the interplay between peptidase and protein is relevant in the search for health-promoting food-derived hydrolysates and bioactive peptides.

### 2.2. Molecular Interactions between DPP-IV and Their Inhibitory Peptides

The molecular docking analysis was validated by performing a redocking of the crystallographic structures of the ligand Omarigliptin (PDB ID: 4PNZ) with the DPP-IV. The RMSD between the best-docked conformation and the original ligand was 0.972 Å. Most molecular interactions of the predicted ligand with the active site of DPP-IV were shared with the crystallographic ligand (Supplementary Material Figure S1). Of the 92 DPP-IV inhibitory peptides, 46 had a tridimensional structure available in PubChem. Table 2 shows the binding energy of these inhibitory peptides with the DPP-IV active site. The binding energy values ranged from −5.2 kcal/mol to −7.9 kcal/mol, a range similar to the one reported for peptides from other food sources (−6.57 to −8.037 kcal/mol) [25,26,27]. The lower binding energy value indicates a higher affinity for the DPP-IV active site [5]. Interestingly, the binding energy values of some chickpea peptides are comparable to the values reported for DPP-IV inhibitor drugs, such as saxagliptin (−8.4 kcal/mol) and vildagliptin (−8.84 kcal/mol) [28,29], highlighting that DPP-IV inhibitory chickpea peptides have a high affinity for the DPP-IV active site, and suggesting that they could be effective competitive DPP-IV inhibitors. Analyses of molecular docking for all the peptides examined in the present study can be found in a freely accessible repository (https://doi.org/10.6084/m9.figshare.23652000.v1 (accessed on 10 July 2023)).

The DPP-IV active site has four pockets (S1, catalytic site, S2, and S2′): the S1 active site, which contains hydrophobic residues (Tyr 547, Tyr 631, Val 656, Trp 659, Tyr 662, Val 711), the catalytic site (CS) (Ser 630, Asp 708, Asn 710, His 740), S2 (Glu 205, Glu 206, Arg 125), and S2′ (Val 207, Ser 209, Arg 358, Phe 357) active sites [30]. Of the 46 peptides (65.2%), 30 could form hydrophobic interactions with the active site of DPP-IV. Of those, 21 (45.65%) and 17 (36.96%) had a hydrophobic amino acid or tyrosine in the N-terminal or C-terminal positions, respectively. DPP-IV inhibitory peptides usually have a hydrophobic or aromatic amino acid at the N-terminal (Ile, Leu, Val, Phe, Trp, or Tyr) [5], which is crucial for forming hydrophobic interactions with the S1 active site [31]. These peptide characteristics can explain the similar binding energy values between the peptides identified in the present study (Table 2) and the drugs saxagliptin (−8.4 kcal/mol) and vildagliptin (−8.84 kcal/mol) [28,29]. 

**Figure 1:**
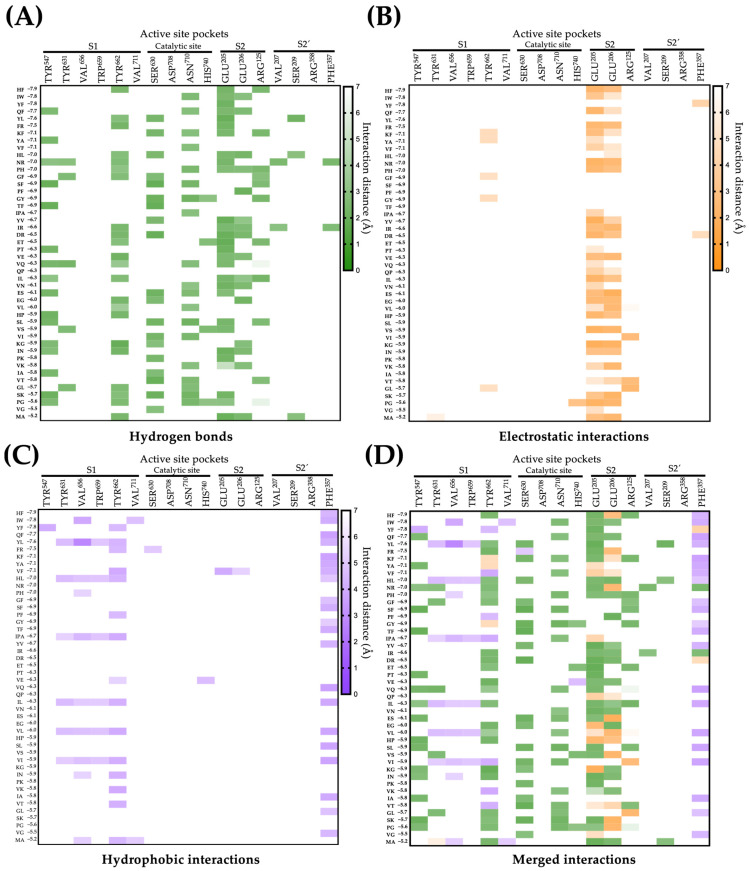
Interactions between chickpea peptides and the active site of DPP-IV. (**A**) Interactions by hydrogen bonds of peptides with the active site of DPP-IV. (**B**) Electrostatic interactions of peptides with the active site of DPP-IV. (**C**) Hydrophobic interactions of peptides with the active site of DPP-IV. (**D**) Merged interactions of peptides with the active site of DPP-IV.

Figure 1 shows the interactions of chickpea peptides with the active sites of DPP-IV (for more details see Supplementary Materials Table S9). Seventeen peptides (36.9%) form hydrophobic interactions with pocket S1 and thirty-two (69.5%) interact with the catalytic pocket through hydrogen bonds. Regarding pockets S2 and S2′, 35 (76.08%) and 21 (45.6%) peptides established electrostatic or hydrophobic interactions, respectively. Pocket S1 is the narrowest and tends to bind small hydrophobic compounds, while pocket S2 accommodates larger compounds through salt bridges [31]. Notably, hydrogen bonds contribute to stabilizing the complex peptide/active site of DPP-IV [32], highlighting an enhanced affinity of the peptides for the active site of DPP-IV.

**Figure 2:**
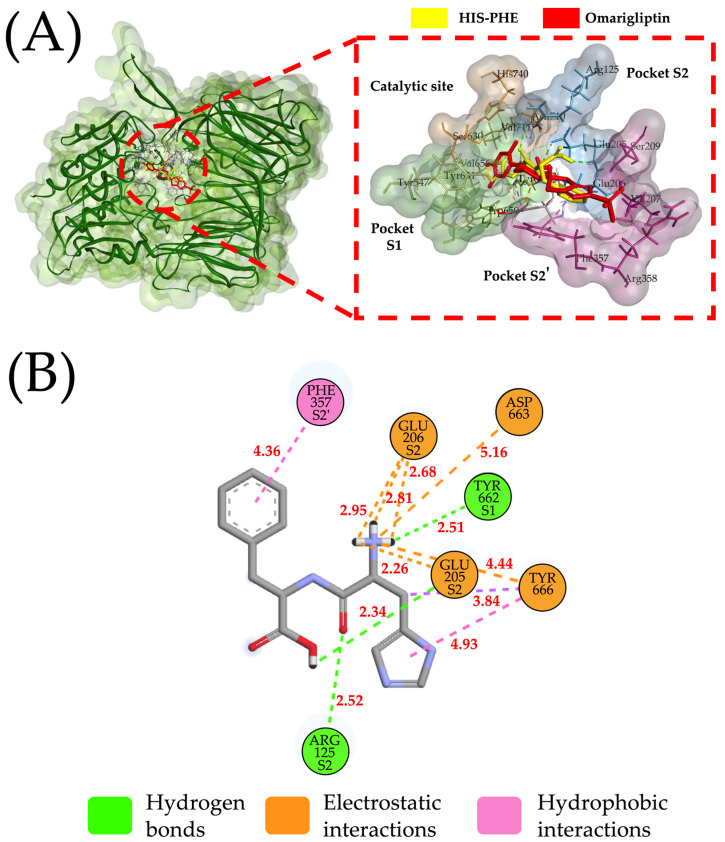
Molecular docking and interactions of the His–Phe peptide with DPP-IV. (**A**) Three-dimensional visualization of the molecular docking of the His–Phe peptide and the reference drug omarigliptin in the active site of DPP-IV (Red dashed lines show a close-up of the active site of DPP-IV). (**B**) Two-dimensional interactions of the His–Phe peptide with the active site of DPP-IV.

The overlapping His–Phe peptide (−7.9 kcal/mol binding energy) in the 3D structure of the complex DPP-IV (PDB: 4PNZ)/omarigliptin is shown in Figure 2A. His–Phe could establish four electrostatic interactions with the residues Glu 205 and Glu 206 of the S2 pocket (Figure 2B). Furthermore, His could interact with the active site residues Arg 125 and Tyr 662 of S2 pocket through strong hydrogen bonds (<3 Å) (Figure 2B). On the other hand, Phe can foster hydrophobic interactions with other non-polar amino acids, enhancing the stability of the complex peptide/active site of DPP-IV. These overall interactions could explain the low binding energy of the complex His–Phe/active site of DPP-IV (−7.9 kcal/mol). Xu et al. [17,18] identified three promising peptides from *Brassica napus* seed proteins using HPLC Triple-TOF MS/MS. The authors stated that the peptides showed prominent inhibitory activity with the energy of affinity values with DPP-IV ranging from −2.21 to −1.76 kcal/mol (−9.27 to −7.38 kJ/mol) and IC50 values from 52.16 to 135.7 µM [17,18]. Others reported a thirteen-amino acid peptide from chickpea proteins, which was identified using liquid chromatography–electrospray ionization–MS/MS [19]. The peptide has an energy of affinity value with the DPP-IV of −7.3 kcal/mol, and according to the authors, chickpea peptides could be used as an ingredient for designing functional foods, or treating or preventing DM2.

### 2.3. ADMET Properties

ADMET helps predict the pharmacokinetics of BPs. Ideally, therapeutic compounds should be bioavailable, adequately distributed, non-toxic, and persist in the organism for enough time to perform their biological effect [33]. Table 3 presents the ADMET values of the 10 DPP-IV inhibitory peptides that have a 3D structure available in PubChem and show the lowest binding energy with the active site of DPP-IV. The specific ADMET values of all the peptides are detailed in the Supplementary Materials (Table S10). The 46 peptides passed the Lipinski rule, suggesting that they could reach systemic circulation (Table 3). Human intestinal absorption and oral bioavailability are relevant factors for novel therapeutic agents. In the present study, 35 (76.09%) and 41 (89.13%) chickpea DPP-IV inhibitory peptides showed optimal human intestinal absorption and bioavailability values, respectively (Table 3).

A high distribution volume suggests that therapeutic peptides can reach the target tissues, potentially increasing their effectiveness [34]. In this context, the 46 identified peptides showed optimal distribution values (Table 3). Furthermore, the peptides’ likelihood of having an extended half-life (>3 h) is promising (Table 3). Contrary, vildagliptin has a half-life of approximately 90 min and saxagliptin of 2.5–3 h. Omarigliptin has a long half-life, mainly due to its strong affinity for plasma proteins such as albumin [35,36,37].

Of the 46 peptides, 45 (97.8%)were non-toxic. The Ile–Trp peptide was the only potentially toxic peptide identified, but it is a potent ACE-I inhibitor that has been reported as a potential therapeutic peptide [38,39]. These facts highlight that ADMET prediction should be considered as a guideline for designing in vitro and in vivo studies.

### 2.4. Multi-Bioactivities and Stability for Gastrointestinal Digestion

Biological activities other than DPP-IV inhibition were evaluated for the 46 identified peptides. Twenty-eight (60.86%) peptides showed ACE-I inhibitory potential and 7 (15.21%) the potential to inhibit renin. DPP-IV inhibitory peptides and ACE-I inhibitory ones share similar characteristics, such as low molecular weight, and usually contain aromatic and/or hydrophobic amino acids [12,25]. This is of relevance since approximately 70% of individuals with diabetes also suffer from hypertension [40]. Additionally, the 46 peptides were subjected to sequential pepsin, chymotrypsin, and trypsin hydrolysis to evaluate their gastrointestinal digestion stability. Thirty-four (73.9%) peptides were stable for such hydrolysis. These results are in line with the fact that di- and tripeptides tend to resist gastrointestinal digestion [12]. Notably, 24 out of 34 peptides showed multi-bioactivity in addition to stability for in silico gastrointestinal digestion.

## 3. Materials and Methods

### 3.1. Protein Sequence, Enzymatic Hydrolyses, and Analyses of DPP-IV Inhibitory Peptides

**Figure 3:**
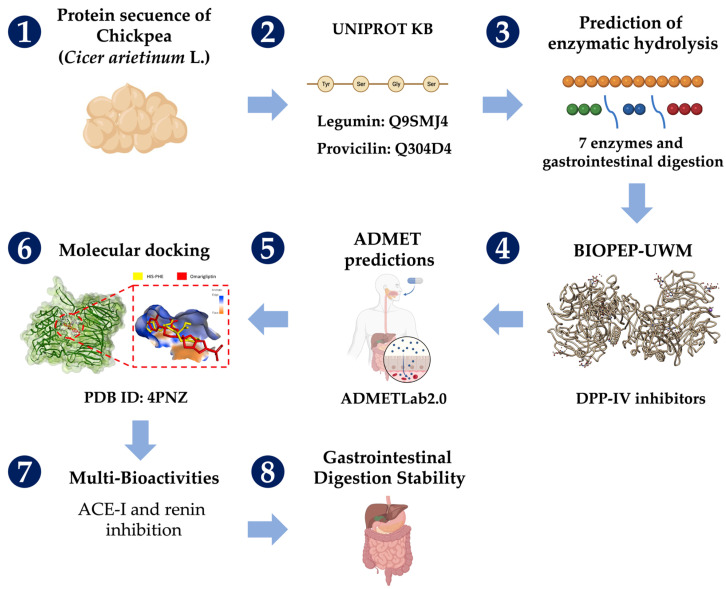
General outline of the methodology (Numbers indicate the order of the analyses performed; red dashed lines show a close-up of the active site of DPP-IV).

Figure 3 outlines the overall methodology employed in this study. The primary structures of chickpea legumin (UniProt ID: Q9SMJ4) and provicilin, (UniProt ID: Q304D4) were obtained (UniProtKB). These proteins are found in high proportion in the globulin fraction of chickpea seed proteins, which account for about 52% of the total chickpea protein content [41]. Hydrolyses were carried out using alcalase (EC 3.4.21.62), papain (3.4.22.2), bromelain (3.4.22.32), ficin (EC 3.4.22.3), pepsin (EC 3.4.23.1), trypsin (EC 3.4.21.4), and chymotrypsin (EC 3.4.21.1) (BIOPEP-UWM) [42]. Gastrointestinal digestion was also carried out (pepsin (EC 3.4.23.1), trypsin (EC 3.4.21.4), and chymotrypsin (EC 3.4.21.1)) (BIOPEP-UWM). The peptides released were screened for DPP-IV inhibitory activity. The frequency of occurrence values (‘A’ (in the proteins) and ‘A_E_’ (in the hydrolysates)) and inhibitory potentials (‘B’ and ‘B_E_’) of each DPP-IV inhibitory peptide were calculated (BIOPEP-UWM). A higher ‘A_E_’ value indicated a larger number of DPP-IV inhibitory peptides released, and the lower the ‘B_E_’ value, the higher the peptide’s DPP-IV inhibitory potential. ADMET (absorption, distribution, metabolism, excretion, and toxicity) pharmacokinetic property prediction and molecular docking analyses [33] were carried out for peptides with a PubChem structure, as described below.

### 3.2. ADMET Predictions

The following pharmacokinetic properties were calculated for each DPP-IV inhibitory peptide using the ADMETLab2.0 platform [33]: (1) Lipinski’s rule; (2) human intestinal absorption (HIA; optimal range (OR): 20–30%); (3) volume of distribution (VD; OR: 0.04–20 L/kg); (4) half-life (t1/2; OR: ≥ 3.0 h); (5) acute oral toxicity in rats (LD50; OR > 500 mg/kg). The results were interpreted following the ADMETLab2.0 platform criteria. Analyses were carried out as described previously [43].

### 3.3. Molecular Docking

The human DPP-IV crystallographic structure, complexed with the inhibitor Omarigliptin (PDB ID: 4PNZ), was obtained from the Protein Data Bank. Since DPP-IV is a homologous dimer, subunit B was utilized for carrying out in silico analyses. The accuracy and reliability of docking analysis were assessed by performing a re-docking of the original crystallographic ligand Omarigliptin (extracted from the exact coordinates of PDB ID: 4PNZ) with the human DPP-IV crystallographic structure. The root mean square deviation (RMSD) between the predicted ligand and the crystallographic ligand (as found in the crystallographic structure) was calculated using the validated DockingRMSD platform (University of Michigan, Ann Arbor, MI, USA.) [44]. An RMSD < 2 Å grid spacing between the best prediction of the docked ligand and the crystallographic ligand was considered acceptable.

DPP-IV inhibitory peptides’ 3D structures were obtained from the PubChem database. Peptides without 3D structures available were excluded. Polar hydrogens and charges were added to the peptides and DPP-IV for performing molecular docking [45]. Additionally, water molecules and the complexed inhibitor were removed from the DPP-IV structure (UCSF Chimera). For determining the coordinates including the DPP-IV active site, a redocking analysis was carried out (AutoDock Vina 1.1.2 flexible docking tool (Scripps Research Institute, San Diego, CA, USA.)) [46]. The coordinates were as follows: x: −6.733; y: 62.839; z: 35.416, with a radius of 20 Å. Molecular docking parameters were set as follows: the number of binding modes per (ligand was 10, the exhaustiveness was 8, and the maximum energy difference between the modes was 2 kcal/mol. The peptides that interact with the DPP-IV active site (visualized using Discovery Studio v21.1.0 (BIOVIA, Dassault Systèmes, San Diego, CA, USA.) in molecular docking were selected based on their binding energy, the lowest binding energy, and the best pose of each inhibitory peptide. Molecular dockings with unfavorable interactions were excluded. 

### 3.4. Multi-Bioactivities and Stability to Gastrointestinal Digestion

For each peptide subjected to molecular docking, bioactivities other than DPP-IV inhibition were assessed, as well as their susceptibility to gastrointestinal digestion (pepsin (EC 3.4.23.1), trypsin (EC 3.4.21.4), and chymotrypsin (EC 3.4.21.1)) (BIOPEP-UWM). 

## 4. Conclusions

Chickpea legumin and provicilin are promising sources of DPP-IV inhibitory peptides. Ninety-two unique peptides can be obtained after the hydrolysis of these chickpea proteins with commonly utilized proteolytic enzymes. The peptides released are di- and tripeptides, and most of them show favorable pharmacokinetic parameters, such as optimal human intestinal absorption and bioavailability values, high distribution volume, extended half-life, and reduced or lack of toxicity. Particularly, the peptides HF and IW showed promising energies of affinity with DPP-IV (−7.9 and −7.8, respectively). The HF peptide interacts with the residues of active sites S1 (TYR662) and S2 (GLU205/ARG125) of DPP-IV through hydrogen bonds (distances < 3.0 Å) and electrostatic interactions with residues of S2 (GLU205/GLU206) (distances < 3.0 Å). Furthermore, HF interacts with the PHE357 residue of the S2′ pocket (4.36 Å) through a hydrophobic interaction. IW interacts with S1 (VAL656/VAL711) and S2′ through hydrophobic interactions (distances < 5.0 A), with the catalytic site (ASN710) and S2 (GLU205/GLU206) through hydrogen bonds (distances < 3 Å), and with S2 (GLU205/GLU206) through electrostatic interactions (distance < 5.5 Å). Additionally, the peptides have the capacity to inhibit ACE-I, showing their antihypertensive potential. Due to the promising pharmacokinetics of the peptides and their energy of affinity with DPP-IV, studies to evaluate the peptides’ DPP-IV inhibitory potential and their impact on insulin and glucose serum levels are warranted. 

## Figures and Tables

**Table 1:** Occurrence and potential of DPP-IV inhibitory peptides in the sequence and hydrolysates of chickpea provicilin and legumin.

Protein	A	B	Enzyme	A_E_	B_E_
Provicilin	0.6093	0.0003526629028523	Pepsin	0.022	4.7635643240156 × 10^−5^
			Chymotrypsin	0.0287	3.2429337219228 × 10^−5^
			Trypsin	0.0088	6.8625336714504 × 10^−7^
			Gastrointestinal	0.0596	6.7698735295912 × 10^−5^
			Papain	0.0795	8.271490429923 × 10^−5^
			Ficin	0.0728	6.4223543298536 × 10^−5^
			Stem bromelain	0.0817	6.0467750947608 × 10^−5^
			Subtilisin (Alcalase)	0.0662	0.000165417
Legumin	0.5847	0.0002483186581999	Pepsin	0.0141	5.3720267147866 × 10^−6^
			Chymotrypsin	0.0423	1.302957714179 × 10^−5^
			Trypsin	0.0040	0
			Gastrointestinal	0.0585	1.8401603856577 × 10^−5^
			Papain	0.0927	2.5172135076017 × 10^−5^
			Ficin	0.0484	4.2932403610376 × 10^−5^
			Stem bromelain	0.0867	0
			Subtilisin (Alcalase)	0.0383	9.1420948488684 × 10^−6^

A = Frequency of DPP-IV inhibitory peptide occurrence in protein sequence; B = Potential DPP-IV activity of the protein; A_E_ = frequency of DPP-IV inhibitory peptide release by selected enzymes; B_E_ = Potential activity of DPP-IV inhibitory peptides released by enzymes. Higher values of A and A_E_ equal a higher frequency of DPP-IV inhibitory peptides. Lower values of B and B_E_ equal higher-potential DPP-IV inhibitory activity of the protein sequence and the peptides released by selected enzymes, respectively.

**Table 2:** DPP-IV inhibitory peptides, binding energy, and enzyme-releasing peptides.

Peptide	BIOPEP ID	Binding Energy (Kcal/Mol)	Protein	Location	Released by	Pubchem ID
HF	8791	−7.9	Provicilin	426–427	Stem bromelain; Subtilisin	152198
			Legumin	473–474	Stem bromelain; Subtilisin; Pepsin; Papain	
IW	8807	−7.8	Legumin	457–458	Subtilisin	7019084
YF	8935	−7.8	Provicilin	95–96	Stem bromelain	7009600
			Legumin	99–100	Stem bromelain; Papain	
QF	8870	−7.7	Provicilin	153–154	Gastrointestinal; Papain; Ficin	57288566
			Legumin	61–62	Gastrointestinal; Papain; Ficin	
YL	8940	−7.6	Legumin	182–183	Pepsin; Papain; Stem bromelain	87071
FR	8780	−7.5	Legumin	135–136	Trypsin	150903
KF	8809	−7.1	Legumin	124–125	Stem bromelain; Subtilisin	151410
VF	8917	−7.1	Provicilin	58–59 122–123	Papain; Subtilisin	6993120
			Legumin	103–104 148–149 403–404	Chymotrypsin; Gastrointestinal; Ficin; Subtilisin	
YA	8932	−7.1	Legumin	384–385 394–395	Stem bromelain	7020632
HL	8557	−7	Provicilin	72–73	Pepsin; Papain; Stem bromelain; Subtilisin	189008
NR	8849	−7	Provicilin	162–163	Papain; Ficin; Stem bromelain	14299174
			Legumin	133–134 225–226	Stem bromelain	
PH	8856	−7	Provicilin	295–296	Chymotrypsin; Gastrointestinal; Ficin	9856353
GF	8782	−6.9	Provicilin	63–64 375–376 377–378	Chymotrypsin; Pepsin; Gastrointestinal; Subtilisin	92953
			Legumin	212–213 312–313	Chymotrypsin; Gastrointestinal; Subtilisin	
GY	8788	−6.9	Legumin	98–99	Chymotrypsin; Gastrointestinal	92829
PF	8854	−6.9	Provicilin	362–363	Chymotrypsin; Gastrointestinal; Ficin	6351946
			Legumin	471–472	Stem bromelain	
SF	8891	−6.9	Provicilin	176–177	Chymotrypsin; Pepsin; Gastrointestinal; Papain	7009597
			Legumin	10–11 347–348	Chymotrypsin; Pepsin; Gastrointestinal; Papain	
TF	8900	−6.9	Provicilin	110–111	Gastrointestinal; Ficin	7010580
IPA	8304	−6.7	Provicilin	357–359	Stem bromelain	10040393
YV	8946	−6.7	Legumin	433–434	Stem bromelain	7009560
DR	8769	−6.6	Provicilin	203–204 416–417	Trypsin; Gastrointestinal; Ficin; Stem bromelain	16122509
			Legumin	440–441 429–430	Gastrointestinal; Ficin; Stem bromelain	
IR	8806	−6.6	Provicilin	442–443	Trypsin; Gastrointestinal; Ficin	7021814
ET	8774	−6.5	Legumin	109–110	Stem bromelain	6998031
PT	8863	−6.5	Legumin	144–145	Stem bromelain	53860028
IL	8802	−6.3	Provicilin	139–140 168–169 304–305 449–450	Gastrointestinal; Papain; Ficin; Stem bromelain; Subtilisin	7019083
			Legumin	382–383	Ficin; Stem bromelain; Subtilisin	
QP	8532	−6.3	Provicilin	431–432	Papain	11736661
			Legumin	25–26		
VE	8916	−6.3	Provicilin	56–57	Subtilisin	7009623
VN	8924	−6.3	Legumin	132–133 224–225	Chymotrypsin; Gastrointestinal	7020201
VQ	8925	−6.3	Provicilin	340–341	Subtilisin	7016045
EG	8770	−6.1	Legumin	48–49 121–122 137–138	Papain; Ficin; Stem bromelain	6427052
ES	8773	−6.1	Provicilin	244–245	Ficin; Subtilisin; Stem bromelain	6995653
			Legumin	116–117	Ficin; Stem bromelain	
HP	8520	−6	Legumin	477–478	Papain	152322
VL	8922	−6	Provicilin	92–93 101–102 104–105 124–125 186–187	Chymotrypsin; Pepsin; Gastrointestinal; Papain; Ficin; Subtilisin	6993117
IN	8804	−5.9	Provicilin	378–379	Papain	7016080
			Legumin	444–445	Chymotrypsin; Gastrointestinal; Subtilisin	
KG	8810	−5.9	Provicilin	311–312	Papain; Stem bromelain	7022320
			Legumin	248–249 387–388	Papain; Stem bromelain	
PK	8858	−5.9	Provicilin	195–196	Trypsin; Gastrointestinal; Ficin	9209431
SL	8560	−5.9	Provicilin	6–7 61–62 128–129 291–292	Chymotrypsin; Pepsin; Gastrointestinal; Papain	7015694
			Legumin	8–9 78–79 364–365 426–427	Chymotrypsin; Pepsin; Gastrointestinal; Papain	
VI	8920	−5.9	Provicilin	217–218	Subtilisin	7010531
VS	8926	−5.9	Provicilin	37–38 67–68 241–242	Ficin; Subtilisin	6992640
			Legumin	162–163	Subtilisin	
IA	8525	−5.8	Provicilin	69–70 166–167	Stem bromelain	7009577
			Legumin	160–161 422–423		
VK	8921	−5.8	Legumin	245–246 247–248	Trypsin; Gastrointestinal; Ficin; Subtilisin	168058
VT	8927	−5.8	Provicilin	120–121	Subtilisin	9815826
			Legumin	345–346	Papain	
GL	8561	−5.7	Provicilin	74–75	Chymotrypsin; Pepsin; Gastrointestinal; Subtilisin	1548899
SK	8894	−5.7	Provicilin	230–231	Gastrointestinal	16122513
PG	8855	−5.6	Provicilin	409–410	Papain; Ficin; Stem bromelain	6426709
			Legumin	105–106 463–464	Papain; Ficin; Stem bromelain	
VG	8918	−5.5	Provicilin	317–318	Papain; Ficin	6993110
MA	3173	−5.2	Legumin	1–2	Stem bromelain	7009581

**Table 3:** ADMET pharmacokinetic properties of gliptins and chickpea-derived DPP-IV inhibitory peptides.

Peptide/Drug	Lipinski Rules	HIA	F 20%	F 30%	VD (L/kg)	T 1/2 (h)	ROAT
Omarigliptin	Accepted	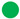	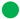	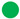	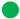	0.151	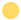
Saxagliptin	Accepted	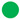	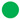	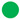	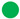	0.309	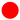
Vildagliptin	Accepted	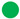	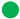	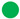	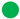	0.37	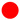
HF	Accepted	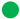	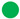	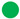	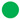	0.92	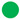
IW	Accepted	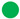	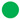	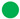	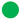	0.91	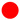
YF	Accepted	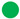	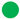	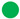	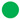	0.921	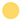
QF	Accepted	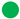	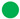	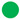	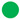	0.61	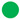
YL	Accepted	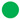	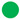	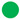	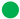	0.909	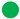
FR	Accepted	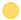	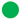	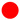	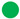	0.826	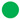
KF	Accepted	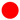	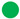	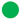	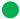	0.835	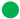
VF	Accepted	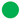	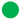	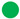	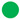	0.872	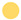
YA	Accepted	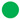	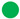	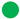	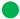	0.894	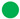
HL	Accepted	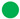	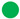	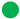	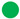	0.919	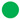
NR	Accepted	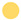	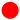	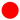	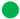	0.422	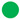
PH	Accepted	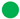	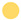	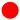	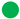	0.887	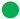

HIA: Human intestinal absorption, F20%: Bioavailability 20%, F30%: Bioavailability 30%, VD: Volume distribution, ROAT: Rat oral acute toxicity. Empirical decision: Green: Excellent, Yellow: Medium, Red: Poor, T1/2: probability to >3H (0–1.0).

## Data Availability

Data are contained within the article and the Supplementary Materials.

## Supplementary Materials

The following supporting information can be downloaded at: https://www.mdpi.com/article/10.3390/ph16081109/s1, Table S1: Peptide release from legumin and provicilin proteins after trypsin hydrolysis; Table S2: Peptide release from legumin and provicilin proteins after pepsin hydrolysis; Table S3: Peptide release from legumin and provicilin proteins after chymotrypsin hydrolysis; Table S4: Peptide release from legumin and provicilin proteins after simulated gastrointestinal digestion; Table S5: Peptide release from legumin and provicilin proteins after papain hydrolysis; Table S6: Peptide release from legumin and provicilin proteins after alcalase hydrolysis; Table S7: Peptide release from legumin and provicilin proteins after ficin hydrolysis; Table S8: Peptide release from legumin and provicilin proteins after stem bromelain hydrolysis; Table S9. Interactions of chickpea peptides with the active site of DPP-IV; Table S10. Predictions of ADMET pharmacokinetics properties of 46 chickpea peptides; Figure S1: Docking validation of the crystallographic structures of Omarigliptin (PDB: 4PNZ) with the human DPP-IV.
