# Supplementary material for: Dipeptidyl Peptidase IV Inhibitory Peptides from Chickpea Proteins (Cicer arietinum L.): Pharmacokinetics, Molecular Interactions, and Multi-Bioactivities
Source: Pharmaceuticals (Basel). 2023 Aug 4;16(8):1109. doi: 10.3390/ph16081109 (PMC10459228; doi:10.3390/ph16081109)
Supplement: Supplementary file 1 [file pharmaceuticals-16-01109-s001.zip › pharmaceuticals-2524987-supplementary.pdf]

Table S1. Peptide release from legumin and provicilin proteins after trypsin hydrolysis.

Protein	Peptide ID	Sequence	Location	Activity	Chemical mass	EC50 uM
Provicilin	8769	DR	[203-204]	Dipeptidyl peptidase IV inhibitor	289.2770	0
Legumin	8780	FR	[135-136]	Dipeptidyl peptidase IV inhibitor	321.3650	0
Provicilin	8806	IR	[442-443]	Dipeptidyl peptidase IV inhibitor	287.3480	0
Provicilin	8858	PK	[195-196]	Dipeptidyl peptidase IV inhibitor	243.2910	0
Legumin	8921	VK	[247-248]	Dipeptidyl peptidase IV inhibitor	245.3070	0

Table S2. Peptide release from legumin and provicilin proteins after pepsin hydrolysis.

Protein	Peptide ID	Sequence	Location	Activity	Chemical mass	EC50 uM
Proviciln	8782	GF	[63-64]	Dipeptidyl peptidase IV inhibitor	222.2290	0
Proviciln	8782	GF	[377-378]	Dipeptidyl peptidase IV inhibitor	222.2290	0
Proviciln	8561	GL	[74-75]	Dipeptidyl peptidase IV inhibitor	188.2120	2615.03
Legumin	8791	HF	[473-474]	Dipeptidyl peptidase IV inhibitor	302.3190	0
Proviciln	8557	HL	[72-73]	Dipeptidyl peptidase IV inhibitor	268.3020	143.19
Proviciln	8891	SF	[176-177]	Dipeptidyl peptidase IV inhibitor	252.2570	0
Legumin	8891	SF	[10-11]	Dipeptidyl peptidase IV inhibitor	252.2570	0
Proviciln	8560	SL	[6-7]	Dipeptidyl peptidase IV inhibitor	218.2400	2517.08
Proviciln	8560	SL	[61-62]	Dipeptidyl peptidase IV inhibitor	218.2400	2517.08
Legumin	8560	SL	[8-9]	Dipeptidyl peptidase IV inhibitor	218.2400	2517.08
Proviciln	8922	VL	[124-125]	Dipeptidyl peptidase IV inhibitor	230.2930	74
Legumin	8940	YL	[182-183]	Dipeptidyl peptidase IV inhibitor	294.3300	0

Table S3. Peptide release from legumin and provicilin proteins after chymotrypsin hydrolysis.

Protein	Peptide ID	Sequence	Location	Activity	Chemical mass	EC50 uM
Provicilin	8782	GF	[63-64]	Dipeptidyl peptidase IV inhibitor	222.2290	0
Provicilin	8782	GF	[375-376]	Dipeptidyl peptidase IV inhibitor	222.2290	0
Legumin	8782	GF	[312-313]	Dipeptidyl peptidase IV inhibitor	222.2290	0
Provicilin	8561	GL	[74-75]	Dipeptidyl peptidase IV inhibitor	188.2120	2615.03
Legumin	8788	GY	[98-99]	Dipeptidyl peptidase IV inhibitor	238.2220	0
Legumin	8804	IN	[444-445]	Dipeptidyl peptidase IV inhibitor	245.2640	0
Provicilin	8854	PF	[362-363]	Dipeptidyl peptidase IV inhibitor	262.2940	0
Provicilin	8856	PH	[295-296]	Dipeptidyl peptidase IV inhibitor	252.2590	0
Provicilin	8891	SF	[176-177]	Dipeptidyl peptidase IV inhibitor	252.2570	0
Legumin	8891	SF	[10-11]	Dipeptidyl peptidase IV inhibitor	252.2570	0
Provicilin	8560	SL	[6-7]	Dipeptidyl peptidase IV inhibitor	218.2400	2517.08
Provicilin	8560	SL	[61-62]	Dipeptidyl peptidase IV inhibitor	218.2400	2517.08
Legumin	8560	SL	[8-9]	Dipeptidyl peptidase IV inhibitor	218.2400	2517.08
Legumin	8560	SL	[78-79]	Dipeptidyl peptidase IV inhibitor	218.2400	2517.08
Legumin	8917	VF	[103-104]	Dipeptidyl peptidase IV inhibitor	264.3100	0
Provicilin	8922	VL	[124-125]	Dipeptidyl peptidase IV inhibitor	230.2930	74
Legumin	8924	VN	[224-225]	Dipeptidyl peptidase IV inhibitor	231.2370	0

Table S4. Peptide release from legumin and provicilin proteins after simulated gastrointestinal digestion.

Protein	Peptide ID	Sequence	Location	Activity	Chemical mass	EC50 uM
Provicilin	8769	DR	[203-204]	Dipeptidyl peptidase IV inhibitor	289.2770	0
Legumin	8769	DR	[440-441]	Dipeptidyl peptidase IV inhibitor	289.2770	0
Provicilin	8782	GF	[63-64]	Dipeptidyl peptidase IV inhibitor	222.2290	0
Provicilin	8782	GF	[375-376]	Dipeptidyl peptidase IV inhibitor	222.2290	0
Legumin	8782	GF	[312-313]	Dipeptidyl peptidase IV inhibitor	222.2290	0
Provicilin	8561	GL	[74-75]	Dipeptidyl peptidase IV inhibitor	188.2120	2615.03
Legumin	8788	GY	[98-99]	Dipeptidyl peptidase IV inhibitor	238.2220	0
Provicilin	8802	IL	[139-140]	Dipeptidyl peptidase IV inhibitor	244.3200	0
Legumin	8804	IN	[444-445]	Dipeptidyl peptidase IV inhibitor	245.2640	0
Provicilin	8806	IR	[442-443]	Dipeptidyl peptidase IV inhibitor	287.3480	0
Provicilin	8854	PF	[362-363]	Dipeptidyl peptidase IV inhibitor	262.2940	0
Provicilin	8856	PH	[295-296]	Dipeptidyl peptidase IV inhibitor	252.2590	0
Provicilin	8858	PK	[195-196]	Dipeptidyl peptidase IV inhibitor	243.2910	0
Provicilin	8870	QF	[153-154]	Dipeptidyl peptidase IV inhibitor	293.3080	0
Legumin	8870	QF	[61-62]	Dipeptidyl peptidase IV inhibitor	293.3080	0
Provicilin	8891	SF	[176-177]	Dipeptidyl peptidase IV inhibitor	252.2570	0
Legumin	8891	SF	[10-11]	Dipeptidyl peptidase IV inhibitor	252.2570	0
Provicilin	8894	SK	[230-231]	Dipeptidyl peptidase IV inhibitor	233.2540	0
Provicilin	8560	SL	[6-7]	Dipeptidyl peptidase IV inhibitor	218.2400	2517.08
Provicilin	8560	SL	[61-62]	Dipeptidyl peptidase IV inhibitor	218.2400	2517.08
Provicilin	8560	SL	[128-129]	Dipeptidyl peptidase IV inhibitor	218.2400	2517.08
Legumin	8560	SL	[8-9]	Dipeptidyl peptidase IV inhibitor	218.2400	2517.08
Legumin	8560	SL	[78-79]	Dipeptidyl peptidase IV inhibitor	218.2400	2517.08
Legumin	8560	SL	[426-427]	Dipeptidyl peptidase IV inhibitor	218.2400	2517.08
Provicilin	8900	TF	[110-111]	Dipeptidyl peptidase IV inhibitor	266.2820	0
Legumin	8917	VF	[103-104]	Dipeptidyl peptidase IV inhibitor	264.3100	0
Legumin	8921	VK	[247-248]	Dipeptidyl peptidase IV inhibitor	245.3070	0
Provicilin	8922	VL	[124-125]	Dipeptidyl peptidase IV inhibitor	230.2930	74
Provicilin	8922	VL	[186-187]	Dipeptidyl peptidase IV inhibitor	230.2930	74
Legumin	8924	VN	[132-133]	Dipeptidyl peptidase IV inhibitor	231.2370	0

Legumin	8924	VN	[224-225]	Dipeptidyl peptidase IV inhibitor	231.2370	0

Table S5. Peptide release from legumin and provicilin proteins after papain hydrolysis.

Protein	Peptide ID	Sequence	Location	Activity	Chemical mass	EC50 uM
Legumin	8770	EG	[121-122]	Dipeptidyl peptidase IV inhibitor	204.1680	0
Legumin	8770	EG	[137-138]	Dipeptidyl peptidase IV inhibitor	204.1680	0
Legumin	8791	HF	[473-474]	Dipeptidyl peptidase IV inhibitor	302.3190	0
Provicilin	8557	HL	[72-73]	Dipeptidyl peptidase IV inhibitor	268.3020	143.19
Legumin	8520	HP	[477-478]	Dipeptidyl peptidase IV inhibitor	252.2590	2820
Provicilin	8802	IL	[139-140]	Dipeptidyl peptidase IV inhibitor	244.3200	0
Provicilin	8804	IN	[378-379]	Dipeptidyl peptidase IV inhibitor	245.2640	0
Provicilin	8810	KG	[311-312]	Dipeptidyl peptidase IV inhibitor	203.2260	0
Legumin	8810	KG	[387-388]	Dipeptidyl peptidase IV inhibitor	203.2260	0
Provicilin	8849	NR	[162-163]	Dipeptidyl peptidase IV inhibitor	288.2920	0
Provicilin	8855	PG	[409-410]	Dipeptidyl peptidase IV inhibitor	172.1690	0
Legumin	8855	PG	[105-106]	Dipeptidyl peptidase IV inhibitor	172.1690	0
Legumin	8855	PG	[463-464]	Dipeptidyl peptidase IV inhibitor	172.1690	0
Provicilin	8870	QF	[153-154]	Dipeptidyl peptidase IV inhibitor	293.3080	0
Legumin	8870	QF	[61-62]	Dipeptidyl peptidase IV inhibitor	293.3080	0
Provicilin	8532	QP	[431-432]	Dipeptidyl peptidase IV inhibitor	243.2480	0
Legumin	8532	QP	[25-26]	Dipeptidyl peptidase IV inhibitor	243.2480	0
Provicilin	8891	SF	[176-177]	Dipeptidyl peptidase IV inhibitor	252.2570	0
Legumin	8891	SF	[10-11]	Dipeptidyl peptidase IV inhibitor	252.2570	0
Legumin	8891	SF	[347-348]	Dipeptidyl peptidase IV inhibitor	252.2570	0
Provicilin	8560	SL	[6-7]	Dipeptidyl peptidase IV inhibitor	218.2400	2517.08
Provicilin	8560	SL	[61-62]	Dipeptidyl peptidase IV inhibitor	218.2400	2517.08
Provicilin	8560	SL	[291-292]	Dipeptidyl peptidase IV inhibitor	218.2400	2517.08
Legumin	8560	SL	[8-9]	Dipeptidyl peptidase IV inhibitor	218.2400	2517.08
Legumin	8560	SL	[364-365]	Dipeptidyl peptidase IV inhibitor	218.2400	2517.08
Provicilin	8917	VF	[122-123]	Dipeptidyl peptidase IV inhibitor	264.3100	0

Provicilin	8918	VG	[317-318]	Dipeptidyl peptidase IV inhibitor	174.1850	0
Provicilin	8922	VL	[124-125]	Dipeptidyl peptidase IV inhibitor	230.2930	74
Provicilin	8922	VL	[186-187]	Dipeptidyl peptidase IV inhibitor	230.2930	74
Legumin	8927	VT	[345-346]	Dipeptidyl peptidase IV inhibitor	218.2380	0
Legumin	8935	YF	[99-100]	Dipeptidyl peptidase IV inhibitor	328.3470	0
Legumin	8940	YL	[182-183]	Dipeptidyl peptidase IV inhibitor	294.3300	0

Table S6. Peptide release from legumin and provicilin proteins after alcalase hydrolysis.

Protein	Peptide ID	Sequence	Location	Activity	Chemical mass	EC50 uM
Provicilin	8773	ES	[244-245]	Dipeptidyl peptidase IV inhibitor	234.1960	0
Legumin	8782	GF	[212-213]	Dipeptidyl peptidase IV inhibitor	222.2290	0
Provicilin	8782	GF	[63-64]	Dipeptidyl peptidase IV inhibitor	222.2290	0
Provicilin	8782	GF	[375-376]	Dipeptidyl peptidase IV inhibitor	222.2290	0
Provicilin	8561	GL	[74-75]	Dipeptidyl peptidase IV inhibitor	188.2120	2615.03
Legumin	8791	HF	[473-474]	Dipeptidyl peptidase IV inhibitor	302.3190	0
Provicilin	8791	HF	[426-427]	Dipeptidyl peptidase IV inhibitor	302.3190	0
Provicilin	8557	HL	[72-73]	Dipeptidyl peptidase IV inhibitor	268.3020	143.19
Legumin	8802	IL	[382-383]	Dipeptidyl peptidase IV inhibitor	244.3200	0
Provicilin	8802	IL	[449-450]	Dipeptidyl peptidase IV inhibitor	244.3200	0
Legumin	8804	IN	[444-445]	Dipeptidyl peptidase IV inhibitor	245.2640	0
Legumin	8807	IW	[457-458]	Dipeptidyl peptidase IV inhibitor	317.3730	0
Legumin	8809	KF	[124-125]	Dipeptidyl peptidase IV inhibitor	293.3510	0
Provicilin	8916	VE	[56-57]	Dipeptidyl peptidase IV inhibitor	246.2490	0
Legumin	8917	VF	[103-104]	Dipeptidyl peptidase IV inhibitor	264.3100	0
Legumin	8917	VF	[148-149]	Dipeptidyl peptidase IV inhibitor	264.3100	0
Legumin	8917	VF	[403-404]	Dipeptidyl peptidase IV inhibitor	264.3100	0
Provicilin	8917	VF	[58-59]	Dipeptidyl peptidase IV inhibitor	264.3100	0
Provicilin	8917	VF	[122-123]	Dipeptidyl peptidase IV inhibitor	264.3100	0
Provicilin	8920	VI	[217-218]	Dipeptidyl peptidase IV inhibitor	230.2930	0
Legumin	8921	VK	[245-246]	Dipeptidyl peptidase IV inhibitor	245.3070	0

Provicilin	8922	VL	[92-93]	Dipeptidyl peptidase IV inhibitor	230.2930	74
Provicilin	8922	VL	[101-102]	Dipeptidyl peptidase IV inhibitor	230.2930	74
Provicilin	8922	VL	[104-105]	Dipeptidyl peptidase IV inhibitor	230.2930	74
Provicilin	8922	VL	[124-125]	Dipeptidyl peptidase IV inhibitor	230.2930	74
Provicilin	8922	VL	[186-187]	Dipeptidyl peptidase IV inhibitor	230.2930	74
Provicilin	8925	VQ	[340-341]	Dipeptidyl peptidase IV inhibitor	245.2640	0
Legumin	8926	VS	[162-163]	Dipeptidyl peptidase IV inhibitor	204.2130	0
Provicilin	8926	VS	[37-38]	Dipeptidyl peptidase IV inhibitor	204.2130	0
Provicilin	8926	VS	[67-68]	Dipeptidyl peptidase IV inhibitor	204.2130	0
Provicilin	8926	VS	[241-242]	Dipeptidyl peptidase IV inhibitor	204.2130	0
Provicilin	8927	VT	[120-121]	Dipeptidyl peptidase IV inhibitor	218.2380	0

Table S7. Peptide release from legumin and provicilin proteins after ficin hydrolysis.

Protein	Peptide ID	Sequence	Location	Activity	Chemical mass	EC50 uM
Provicilin	8769	DR	[203-204]	Dipeptidyl peptidase IV inhibitor	289.2770	0
Legumin	8769	DR	[429-430]	Dipeptidyl peptidase IV inhibitor	289.2770	0
Legumin	8770	EG	[48-49]	Dipeptidyl peptidase IV inhibitor	204.1680	0
Legumin	8770	EG	[121-122]	Dipeptidyl peptidase IV inhibitor	204.1680	0
Legumin	8770	EG	[137-138]	Dipeptidyl peptidase IV inhibitor	204.1680	0
Provicilin	8773	ES	[244-245]	Dipeptidyl peptidase IV inhibitor	234.1960	0
Legumin	8773	ES	[116-117]	Dipeptidyl peptidase IV inhibitor	234.1960	0
Provicilin	8802	IL	[139-140]	Dipeptidyl peptidase IV inhibitor	244.3200	0
Provicilin	8802	IL	[449-450]	Dipeptidyl peptidase IV inhibitor	244.3200	0
Legumin	8802	IL	[382-383]	Dipeptidyl peptidase IV inhibitor	244.3200	0
Provicilin	8806	IR	[442-443]	Dipeptidyl peptidase IV inhibitor	287.3480	0
Provicilin	8849	NR	[162-163]	Dipeptidyl peptidase IV inhibitor	288.2920	0
Provicilin	8854	PF	[362-363]	Dipeptidyl peptidase IV inhibitor	262.2940	0
Provicilin	8855	PG	[409-410]	Dipeptidyl peptidase IV inhibitor	172.1690	0
Legumin	8855	PG	[105-106]	Dipeptidyl peptidase IV inhibitor	172.1690	0
Legumin	8855	PG	[463-464]	Dipeptidyl peptidase IV inhibitor	172.1690	0
Provicilin	8856	PH	[295-296]	Dipeptidyl peptidase IV inhibitor	252.2590	0
Provicilin	8858	PK	[195-196]	Dipeptidyl peptidase IV inhibitor	243.2910	0

Provicilin	8870	QF	[153-154]	Dipeptidyl peptidase IV inhibitor	293.3080	0
Legumin	8870	QF	[61-62]	Dipeptidyl peptidase IV inhibitor	293.3080	0
Provicilin	8900	TF	[110-111]	Dipeptidyl peptidase IV inhibitor	266.2820	0
Legumin	8917	VF	[403-404]	Dipeptidyl peptidase IV inhibitor	264.3100	0
Provicilin	8918	VG	[317-318]	Dipeptidyl peptidase IV inhibitor	174.1850	0
Legumin	8921	VK	[247-248]	Dipeptidyl peptidase IV inhibitor	245.3070	0
Provicilin	8922	VL	[124-125]	Dipeptidyl peptidase IV inhibitor	230.2930	74
Provicilin	8922	VL	[186-187]	Dipeptidyl peptidase IV inhibitor	230.2930	74
Provicilin	8926	VS	[37-38]	Dipeptidyl peptidase IV inhibitor	204.2130	0
Provicilin	8926	VS	[241-242]	Dipeptidyl peptidase IV inhibitor	204.2130	0

Table S8. Peptide release from legumin and provicilin proteins after stem bromelain hydrolysis.

Protein	Peptide ID	Sequence	Location	Activity	Chemical mass	EC50 uM
Provicilin	8769	DR	[416-417]	Dipeptidyl peptidase IV inhibitor	289.2770	0
Legumin	8769	DR	[429-430]	Dipeptidyl peptidase IV inhibitor	289.2770	0
Legumin	8770	EG	[48-49]	Dipeptidyl peptidase IV inhibitor	204.1680	0
Legumin	8770	EG	[121-122]	Dipeptidyl peptidase IV inhibitor	204.1680	0
Legumin	8770	EG	[137-138]	Dipeptidyl peptidase IV inhibitor	204.1680	0
Provicilin	8773	ES	[244-245]	Dipeptidyl peptidase IV inhibitor	234.1960	0
Legumin	8773	ES	[116-117]	Dipeptidyl peptidase IV inhibitor	234.1960	0
Legumin	8774	ET	[109-110]	Dipeptidyl peptidase IV inhibitor	248.2210	0
Provicilin	8791	HF	[426-427]	Dipeptidyl peptidase IV inhibitor	302.3190	0
Legumin	8791	HF	[473-474]	Dipeptidyl peptidase IV inhibitor	302.3190	0
Provicilin	8557	HL	[72-73]	Dipeptidyl peptidase IV inhibitor	268.3020	143.19
Provicilin	8525	IA	[69-70]	Dipeptidyl peptidase IV inhibitor	202.2390	0
Provicilin	8525	IA	[166-167]	Dipeptidyl peptidase IV inhibitor	202.2390	0
Legumin	8525	IA	[160-161]	Dipeptidyl peptidase IV inhibitor	202.2390	0
Legumin	8525	IA	[422-423]	Dipeptidyl peptidase IV inhibitor	202.2390	0
Provicilin	8802	IL	[139-140]	Dipeptidyl peptidase IV inhibitor	244.3200	0
Provicilin	8802	IL	[168-169]	Dipeptidyl peptidase IV inhibitor	244.3200	0
Provicilin	8802	IL	[304-305]	Dipeptidyl peptidase IV inhibitor	244.3200	0

Provicilin	8802	IL	[449-450]	Dipeptidyl peptidase IV inhibitor	244.3200	0
Legumin	8802	IL	[382-383]	Dipeptidyl peptidase IV inhibitor	244.3200	0
Provicilin	8304	IPA	[357-359]	Dipeptidyl peptidase IV inhibitor	299.3560	49
Legumin	8809	KF	[124-125]	Dipeptidyl peptidase IV inhibitor	293.3510	0
Provicilin	8810	KG	[311-312]	Dipeptidyl peptidase IV inhibitor	203.2260	0
Legumin	8810	KG	[248-249]	Dipeptidyl peptidase IV inhibitor	203.2260	0
Legumin	8810	KG	[387-388]	Dipeptidyl peptidase IV inhibitor	203.2260	0
Legumin	3173	MA	[1-2]	Dipeptidyl peptidase IV inhibitor	220.2770	0
Provicilin	8849	NR	[162-163]	Dipeptidyl peptidase IV inhibitor	288.2920	0
Legumin	8849	NR	[133-134]	Dipeptidyl peptidase IV inhibitor	288.2920	0
Legumin	8849	NR	[225-226]	Dipeptidyl peptidase IV inhibitor	288.2920	0
Legumin	8854	PF	[471-472]	Dipeptidyl peptidase IV inhibitor	262.2940	0
Provicilin	8855	PG	[409-410]	Dipeptidyl peptidase IV inhibitor	172.1690	0
Legumin	8855	PG	[105-106]	Dipeptidyl peptidase IV inhibitor	172.1690	0
Legumin	8855	PG	[463-464]	Dipeptidyl peptidase IV inhibitor	172.1690	0
Legumin	8863	PT	[144-145]	Dipeptidyl peptidase IV inhibitor	216.2220	0
Legumin	8932	YA	[384-385]	Dipeptidyl peptidase IV inhibitor	252.2490	0
Legumin	8932	YA	[394-395]	Dipeptidyl peptidase IV inhibitor	252.2490	0
Provicilin	8935	YF	[95-96]	Dipeptidyl peptidase IV inhibitor	328.3470	0
Legumin	8935	YF	[99-100]	Dipeptidyl peptidase IV inhibitor	328.3470	0
Legumin	8940	YL	[182-183]	Dipeptidyl peptidase IV inhibitor	294.3300	0
Legumin	8946	YV	[433-434]	Dipeptidyl peptidase IV inhibitor	280.3030	0

Table S9. Interactions of chickpea peptides with the active site of DPP-IV.

	DPP-IV Active Site																
	Pocket S1						Catalytic triad				Pocket S2			Pocket S2`			
Peptide - Docking	TYR 547	TYR 631	VAL 656	TRP 659	TYR 662	VAL 711	SER 630	ASP 708	ASN 710	HIS 740	GLU 205	GLU 206	ARG 125	VAL 207	SER 209	ARG 358	PHE 357
HL		LEU 2 - Hydophobic (4.58 Å)	LEU 2 - Hydrophobic (4.85 Å)	LEU 2 - Hydrophobic (4.90 Å)	LEU 2 - Hydrogen bond (2.78 Å)<br>LEU 2 - Hydrophobic (4.33 Å)<br>LEU 2 - Hydrophobic (4.73 Å)		LEU 2 - Hydrogen bond (2.50 Å)				HIS 1 - Hydrogen bond (3.08 Å)<br>LEU 2 - Hydrogen bond (2.49 Å)	HIS 1 - Hydrogen bond (2.35 Å)<br>HIS 1- Atractive charge (5.56 Å)			HIS 1 - Hydrogen bond (2.92 Å)		HIS 1 - Hydrophobic (4.91 Å)
SL	SER 1 - Hydrogen bond (2.65 Å)<br>SER 1 - Hydrogen bond (2.35 Å)						SER 1 - Hydrogen bond (2.57 Å)<br>SER 1 - Hydrogen bond (2.22 Å)		SER 1 - Hydrogen bond (2.20 Å)		SER 1 - Hydrogen bond (2.40 Å)		SER 1 - Hydrogen bond (2.60 Å)				LEU 2 - Hydrophobic (3.79Å)
GL		GLY 1 - Hydrogen bond (2.64 Å)			LYS 2 - Hydrogen bond (2.6 Å)<br>GLY 1 - Salt bridge (4.91 Å)				LYS 2 - Hydrogen bond (2.68 Å)				LYS 2 - Salt bridge (2.41 Å)				LYS 2 - Hydrofobic (4.75 Å)
GF		GLY 1 - Hydroge			PHE 2 - Hydroge		PHE 2 -						PHE 2 -				PHE 2 - Hydrofo

		n bond (2.61 Å)			n bond (2.73 Å)<br>GLY 1 - Atractive charge (4.95 Å)	Hydrogen bond (2.63 Å)				Hydrogen bond (2.89 Å)		bic (4.74 Å)
SF	SER 1 - Hydrogen bond (1.95 Å)					SER 1 - Hydrogen bond (1.88 Å)	SER 1 - Hydrogen bond (2.33 Å)			SER 1 - Hydrogen bond (2.64 Å)		PHE 2 - Hydrofobic (4.07 Å)
VL		LEU 2 - Hydrophobic (4.62 Å)	LEU 2 - Hydrophobic (4.74 Å)	LEU 2 - Hydrophobic (4.81 Å)	LEU 2 - Hydrogen bond (3.68 Å)<br>LEU 2 - Hydrophobic (3.99 Å)<br>LEU 2 - Hydrophobic (4.64 Å)		LEU 2 - Hydrogen bond (3.02 Å)	VAL 1 - Atractive charge (5.11 Å)	VAL 1 - Atractive charge (2.44 Å)<br>VAL 1 - Atractive charge (2.95 Å)	LEU 2 - Atractive charge (6.53 Å)		VAL 1 - Hydrophobic (3.81 Å)
HF					HIS 1 - Hydrogen bond (2.51 Å)			HIS 1 - Atractive charge (2.26 Å)<br>PHE 2 - Hydrogen bond (2.34 Å)	HIS 1 - Atractive charge (2.81 Å)<br>HIS 1 - Atractive charge (2.95 Å)<br>HIS 1 - Atractive charge (2.68 Å)	HIS 1 - Hydrogen bond (2.52 Å)		PHE 2 - Hydrophobic (4.36 Å)
YL		LEU 2 - Hydroph	LEU 2 - Hydroph	LEU 2 - Hydroph	LEU 2 - Hidroge	LEU 2 -		LEU 2 -			TYR 1 -	TYR 1 - Hydroph

		obic (4.59 Å)	obic (2.77 Å)	obic (4.86 Å)	n bond (2.77 Å) LEU 2 - Hydrophobic (4.33 Å) LEU 2- Hydrophobic (4.70 Å)		Hydrogen bond (2.40 Å)		Hidrogen bond (2.00 Å)		Hydrogen bond (2.20 Å)	obic (4.21 Å)
ES	GLU 1 - Hydrogen bond (2.34 Å) SER 2 - Hydrogen bond (2.58 Å)						SER 2 - Hydrogen bond (2.16 Å)	SER 2 - Hydrogen bond (2.59 Å)	GLU 1 - Hydrogen bond (2.68 Å) GLU 1 - Atractive charge (2.69 Å) GLU 1 - Atractive charge (2.58 Å) SER 2 - Hydrogen bond (2.97 Å)	GLU 1 - Atractive charge (1.92 Å)		
IW			ILE 1 - Hydrophobic (3.91 Å)			ILE 1- Hydrophobic (4.73 Å)		ILE 1 - Hydrogen bond (2.64 Å)	ILE 1 - Atractive charge (3.28 Å) TRP 2 - Hydrogen	ILE 1 - Atractive charge (5.32 Å) TRP 2 - Hydrogen		TRP 2 - Hydrophobic (4.06 Å) TRP 2 - Hydrophobic (4.96 Å)

							bond (2.50 Å)	bond (2.91 Å)		
KF			LYS 1 - Atractive charge (4.67 Å)	LYS 1 - Hydro gen bond (2.40 Å)	LYS - 1 Hydro gen bond (2.81 Å)		PHE 2 - Hydro gen bond (2.48 Å) LYS 1 - Atracti ve charge (2.75 Å) LYS 1 - Atracti ve charge (2.98 Å)	LYS 1 - Atracti ve charge (4.99 Å)	LYS 1 - Hydro gen bond (2.48 Å)	PHE 2 - Hydroph obic (3.67 Å)
VE			VAL 1 - Hydroge n bond (2.18 Å) VAL 1 - Hydroph obic (5.38 Å)			VAL 1 - Hydroph obic (4.63 Å)	GLU 2 - Hydro gen bond (2.56 Å) VAL 1 - Atracti ve charge (2.75 Å)	GLU 2 - Hydro gen bond (2.69 Å) VAL 1 - Atracti ve charge (2.53 Å) VAL 1 - Atracti ve charge (2.84 Å)		
VQ	VAL 1 - Hydrogen bond (2.41 Å)	GLN 2 - Hydroge	GLN 2 - Hydroge		GLN 2 - Hydro		VAL 1 - Atracti		GLN 2 - Hydro	VAL 1 - Hydroph

	GLN 2 - Hydrogen bond (2.73 Å)	n bond (2.31 Å)			n bond (3.42 Å)		gen bond (2.75 Å)		ve charge (5.18 Å)<br>VAL 1 - Hydrogen bond (2.49 Å)		gen bond (6.39 Å)	obic (3.65 Å)
VS		SER 2 - Hydrogen bond (2.61 Å)						SER 2 - Hydrogen bond (2.6 Å)	VAL 1 - Atractive charge (2.12 Å)<br>VAL 1 - Hydrogen bond (2.52 Å)	VAL 1 - Atractive charge (2.34 Å)		
VI		ILE 2 - Hydrophobic (5.15 Å)	ILE 2 - Hydrophobic (4.77 Å)	ILE 2 - Hydrophobic (4.95 Å)	ILE 2 - Hydrophobic (4.14 Å)	ILE 2 - Hydrogen bond (2.34 Å)	ILE 2 - Hydrogen bond (2.48 Å)				ILE 2 - Atractive charge (2.64 Å)	VAL 1 - Hydrophobic (3.78 Å)
VK					VAL 1 - Hydrophobic (3.97 Å)		VAL 1 - Hydrogen bond (2.91 Å)		VAL 1 - Atractive charge (4.44 Å)<br>LYS 2 - Atractive charge (5.29 Å)<br>LYS 2 - Hydro	LYS 2 - Atractive charge (2.41 Å)<br>LYS 2 - Hydrogen bond (2.78 Å)		

			gen bond (4.92 Å)	
ET	THR 2 - Hydrogen bond	THR 2 - Hydrogen bond (2.72 Å)	GLU 1 - Hydrogen bond (1.91 Å)<br>THR 2 - Hydrogen bond (2.53 Å)	
IA	ALA 2 - Hydrogen bond (2.43 Å)	ALA 2 - Hydrogen bond (2.67 Å)		ILE 1 - Hydrophobic (3.95 Å)
IPA	ILE 1 - Hydrophobic (5.29 Å)<br>ILE 1 - Hydrophobic (4.38 Å)<br>ILE 1 - Hydrophobic (4.84 Å)<br>ILE 1 - Hydrophobic (4.00 Å)	ILE 1 - Hydrogen bond (2.89 Å)	ILE 1 - Atractive charge (4.28 Å)	
MA	MET 1 - Atractive charge (6.00 Å)<br>MET 1 - Hydrophobic (5.22 Å)<br>MET 1 - Hydrophobic (4.39 Å)<br>MET 1 - Hydrogen bond (2.42 Å)<br>MET 1 - Hydrophobic (5.15 Å)		MET 1 - Atractive charge (2.91 Å)<br>ALA 2 - Hydrogen bond (2.04)<br>MET 1 - Atractive charge (2.65 Å)<br>MET 1 - Atractive charge (2.41 Å)<br>MET 1 - Hydrogen bond (2.48 Å)	ALA 2 - Hydrogen bond (2.90 Å)

PT	PRO 1 - Hydrogen bond (3.54 Å)<br>THR 2 - Hydrogen bond (2.34 Å)				PRO 1 - Atractive charge (5.53 Å)<br>THR 2 - Hydrogen bond (2.13 Å)			
YA	ALA 2 - Hydrogen bond (2.43 Å)		ALA 2 - Atractive charge (4.70 Å)		TYR 1 - Atractive charge (4.92 Å)			TYR 1 - Hydrophobic (3.94 Å)
YV				VAL 2 - Hydrogen bond (2.53 Å)	TYR 1 - Atractive charge (1.88 Å)<br>VAL 2 - Hydrogen bond (2.02 Å)	TYR 1 - Atractive charge (4.09 Å)<br>TYR 1 - Hydrogen bond (3.20 Å)		TYR 1 - Hydrophobic (4.28 Å)
NR	ASN 1 - Hydrogen bond (3.02 Å)	ASN 1 - Hydrogen bond (2.93 Å)	ASN 1 - Hydrogen bond (2.67 Å)<br>ASN 1 - Hydrogen bond (2.84 Å)		ARG 2 - Hydrogen bond (2.41 Å)<br>ARG 2 - Hydrogen	ARG 2 - Atractive charge (4.38 Å)<br>ASN 1 - Atractive	ARG 2 - Hydrogen bond (2.49 Å)	ARG 2 - Hydrogen bond (2.45 Å)

					bond (2.60 Å) ASN 1 - Atractive charge (2.15 Å)	charge (2.83 Å) ASN 1 - Atractive charge (2.45 Å)	
SK	SER 1 - Hydrogen bond (2.06 Å)	SER 1 - Hydrogen bond (2.76 Å)	SER 1 - Hydrogen bond (2.08 Å)	SER 1 - Hydrogen bond (2.22 Å)	LYS 2 - Hydrogen bond (2.39 Å) LYS 2 - Atractive charge (3.17 Å) SER 1 - Hydrogen bond (2.22 Å)	LYS 2 - Atractive charge (2.17 Å)	
TF	THR 1 - Hydrogen bond (1.99 Å)		THR 1 - Hydrogen bond (1.93 Å)				PHE 2 - Hydrophobic (4.02 Å)
EG		GLU 1 - Hydrogen bond (2.41 Å)	GLU 1 - Hydrogen bond (2.57 Å)		GLU 1 - Atractive charge (2.56 Å) GLU 1 - Atractive	GLY 2 - Hydrogen bond (2.73 Å) GLY 2 - Hydrogen	

							charge (2.80 Å)	bond (2.23 Å)<br>GLU 1 - Atractive charge (2.51 Å)<br>GLU 1 - Atractive charge (2.51 Å)		
HP	PRO 2 - Hydrogen bond (2.15 Å)				HIS 1 - Hydrogen bond (2.39 Å)		HIS 1 - Atractive charge (2.38 Å)<br>HIS 1 - Atractive charge (2.68 Å)	HIS 1 - Atractive charge (2.98 Å)<br>HIS 1 - Atractive charge (2.97 Å)<br>HIS 1 - Atractive charge (5.04 Å)		
IL	ILE 1 - Hydrogen bond (3.09 Å)	LEU 2 - Hydrophobic (4.55 Å)	LEU 2 - Hydrophobic (5.07 Å)	LEU 2 - Hydrophobic (5.10 Å)	LEU 2 - Hydrogen bond (2.70 Å)<br>LEU 2 - Hydrophobic (4.19 Å)<br>LEU 2 - Hydroph		ILE 1 - Atractive charge (2.22 Å)<br>ILE 1 Hydrogen bond	ILE 1 - Atractive charge (3.09 Å)<br>ILE 1 - Hydrogen bond	LEU 2 - Hydrogen bond (2.31 Å)	ILE 1 - Hydrophobic (3.90 Å)

			obic (4.86 Å)		(2.41 Å)	(3.16 Å)	
IN	ILE 1 - Hydrogen bond (2.71 Å)	ILE 1 - Hydrophobic (5.37 Å)	ILE 1 - Hydrogen bond (2.32 Å) ILE 1 - Hydrophobic (4.18 Å)		ASN 2 - Hydrogen bond (2.12 Å) ASN 2 - Hydrogen bond (2.36 Å) ASN 2 - Hydrogen bond (2.62 Å) ILE 1 - Atractive charge (2.99 Å)	ILE 1 - Atractive charge (2.96 Å) ILE 1 - Atractive charge (4.64 Å) ASN 2 - Hydrogen bond (2.55 Å)	
KG	LYS 1 - Hydrogen bond (2.58 Å)		GLY 2 - Hydrogen bond (2.39 Å) LYS 1 - Hydrogen bond (1.92 Å)	GLY 2 - Hydrogen bond (2.82 Å)	LYS 1 - Atractive charge (1.90 Å)	LYS 1 - Atractive charge (2.08 Å) LYS 1 - Atractive charge (2.35 Å) LYS 1 - Atractive charge (2.64	

						Å) LYS 1 - Hydrogen bond (2.79 Å)		
PG	PRO 1 - Hydrogen bond (2.58 Å)	GLY 2 - Hydrogen bond (2.60 Å)	GLY 2 - Hydrogen bond (2.60 Å) GLY 2 - Hydrogen bond (2.43 Å)	GLY 2 - Hydrogen bond (3.07 Å)	PRO 1 - Hydrogen bond (3.05 Å) PRO 1 - Atractive charge (2.07 Å)	PRO 1 - Atractive charge (2.34 Å)	GLY 2 - Hydrogen bond (6.14 Å)	
QF	GLN 1 - Hydrogen bond (2.57 Å)		GLN 1 - Hydrogen bond (2.82 Å)		PHE 2 - Hydrogen bond (2.50 Å) PHE 2 - Hydrogen bond (2.88 Å) GLN 1 - Atractive charge (2.37 Å)	PHE 2 - Hydrogen bond (2.64 Å) GLN 1 - Atractive charge (4.89 Å)		PHE 2 - Hydrophobic (3.67 Å)
QP					GLN 1 - Atractive	GLN 1 - Atractive		

VF		VAL 1 - Hydrophobic (3.97 Å)		VAL 1 - Hydrogen bond (2.81 Å)		charge (4.01 Å)<br>VAL 1 - Atractive charge (4.00 Å)	charge (5.41 Å)<br>VAL 1 - Atractive charge (5.30 Å)		PHE 2 - Hydrophobic (4.17 Å)
GY		GLY 1 - Atractive charge (4.70 Å)	GLY 1 - Hydrogen bond (2.10 Å)<br>TYR 2 - Hydrogen bond (2.69 Å)	TYR 2 - Hydrogen bond (2.90 Å)<br>TYR 2 - Hydrogen bond (2.20 Å)	TYR 2 - Hydrogen bond (3.01 Å)			TYR 2 - Hydrogen bond (2.43 Å)<br>TYR 2 - Hydrogen bond (2.59 Å)	TYR 2 - Hydrohobic (5.13 Å)
PF		PHE 2 - Hydrophobic (4.52 Å)					PHE 2 - Hydrogen bond (2.09 Å)		
PH	HIS 2 - Hydrophobic (5.40 Å)	HIS 2 - Hydrogen bond (3.08 Å)		HIS 2 - Hydrogen bond (2.49 Å)		HIS 2 - Hydrogen bond (2.69 Å)<br>PHE 1 - Hydrogen bond (2.82 Å)<br>PHE 1 -	PHE 1 - Hydrogen bond (2.75 Å)<br>PHE 1 - Atractive charge (2.87 Å)	HIS 2 - Hydrogen bond (2.57 Å)<br>HIS 2 - Hydrogen bond (2.49 Å)	

VN		VAL 1 - Hydrogen bond (3.08 Å)	Hydrogen bond (2.18 Å)<br>PHE 1 - Atractive charge (2.40 Å)<br>VAL 1 - Atractive charge (4.53 Å)<br>ASN 2 - Hydrogen bond (2.19 Å)<br>ASN 2 - Hydrogen bond (2.18 Å)	ASN 2 - Hydrogen bond (2.54 Å)		
IR	ILE 1 - Hydrogen bond (2.48 Å)<br>ILE 1 - Hydrogen bond (2.78 Å)		ILE 1 - Atractive charge (2.71 Å)<br>ARG 2 - Hydrogen bond (2.59 Å)	ILE 1 - Atractive charge (2.39 Å)<br>ILE 1 - Atractive charge (2.61 Å)<br>ARG 2	ARG 2 - Hydrogen bond (2.95 Å)	ARG 2 - Hydrogen bond (2.67 Å)

					ARG 2 - Hydrogen bond (2.52 Å)	- Atractive charge (4.64 Å)<br>ARG 2 - Hydrogen bond (2.61 Å)		
PK			LYS 2 - Hydrogen bond (2.43 Å)		LYS 2 - Hydrogen bond (2.23 Å)			
VG			GLY 2 - Hydrogen bond (2.87 Å)		VAL 1 - Atractive charge (4.96 Å)			VAL 1 - Hydrophobic (4.37 Å)
VT		VAL 1 - Hydrophobic (4.22 Å)	VAL 1 - Hydrogen bond (2.35 Å)<br>VAL 1 - Hydrogen bond (2.00 Å)	VAL 1 - Hydrogen bond (2.59 Å)	THR 2 - Atractive charge (5.46 Å)	THR 2 - Atractive charge (4.33 Å)	VAL 1 - Hydrogen bond (2.71 Å)<br>VAL 1 - Hydrogen bond (3.04 Å)	
YF	TYR 1 - Hydrophobic (4.03 Å)	PHE 2 - Hydrophobic (4.52 Å)			PHE 2 - Hydrogen bond			TYR 1 - Atractive charge (4.12 Å)

DR	ASP 1 - Hydrogen bond (2.02 Å)	ASP 1 - Hydrogen bond (1.97 Å)	(2.16 Å)<br>ASP 1 - Atractive charge (2.25 Å)<br>ARG - Atractive charge (5.20 Å)	ASP 1 - Atractive charge (5.47 Å)<br>ARG 2 - Atracctive charge (3.07 Å)<br>ARG 2 - Hydrogen bond (2.37 Å)<br>ARG 2 - Hydrogen bond (2.48 Å)	ARG 2 - Atractive charge (4.71 Å)

Table S10. Predictions of ADMET pharmacokinetics properties of 46 chickpea peptides.

Peptide/Drug	Lipinski rules	HIA	$F_{20\%}$	$F_{30\%}$	VD (L/kg)	$T_{1/2}$ < 3 hours	ROAT (mg/kg)
	MW<=500; logP<=5; Hacc<=10; Hdon<=5	HIA<30%= + ; HIA>30%= -	≥20%: F20- ; <20%: F20+	≥30%: F30- ; <30%: F30+	0.04-20	0–0.3 excellent (green, < 3 H) 0.3–0.7 medium (yellow) 0.7–1.0 poor (red, > 3 H)	> 500
Omarigliptin	Accepted	(---)	(---)	(---)	1.324	0.151	(-)
Saxagliptin	Accepted	(---)	(---)	(---)	1.261	0.309	(+++)
Vildagliptin	Accepted	(---)	(---)	(---)	1.063	0.37	(+++)
DR	Accepted	(+++)	(++)	(-)	0.819	0.542	(---)
EG	Accepted	(---)	(---)	(---)	0.279	0.8	(---)
ES	Accepted	(-)	(---)	(-)	0.453	0.884	(---)
ET	Accepted	(---)	(---)	(---)	0.302	0.929	(---)
FR	Accepted	(+)	(--)	(++)	0.35	0.826	(--)
GF	Accepted	(--)	(---)	(---)	0.284	0.886	(---)
GL	Accepted	(---)	(---)	(---)	0.446	0.874	(---)
GY	Accepted	(---)	(--)	(---)	0.357	0.895	(---)
HF	Accepted	(--)	(---)	(---)	0.323	0.92	(--)
HL	Accepted	(---)	(---)	(---)	0.402	0.919	(--)
HP	Accepted	(---)	(---)	(---)	0.384	0.907	(---)
IA	Accepted	(---)	(---)	(---)	0.35	0.849	(---)
IL	Accepted	(---)	(---)	(---)	0.39	0.851	(-)
IN	Accepted	(---)	(---)	(---)	0.333	0.52	(---)
IPA	Accepted	(+)	(---)	(---)	0.336	0.854	(---)
IR	Accepted	(---)	(---)	(---)	0.532	0.384	(--)
IW	Accepted	(---)	(---)	(---)	0.255	0.91	(++)
KF	Accepted	(++)	(---)	(---)	0.415	0.835	(--)
KG	Accepted	(--)	(---)	(---)	0.619	0.709	(---)
MA	Accepted	(---)	(---)	(---)	0.368	0.886	(---)
NR	Accepted	(+)	(+++)	(++)	0.788	0.422	(---)
PF	Accepted	(-)	(+)	(+++)	0.461	0.823	(-)
PG	Accepted	(+)	(--)	(-)	0.609	0.766	(---)
PH	Accepted	(---)	(+)	(+++)	0.5	0.887	(--)
PK	Accepted	(++)	(-)	(++)	0.723	0.739	(---)
PT	Accepted	(---)	(--)	(---)	0.596	0.881	(---)
QF	Accepted	(--)	(---)	(---)	0.249	0.61	(---)
QP	Accepted	(---)	(---)	(---)	0.32	0.491	(---)
SF	Accepted	(+)	(---)	(---)	0.683	0.805	(---)
SK	Accepted	(++)	(---)	(--)	0.558	0.829	(---)
SL	Accepted	(---)	(---)	(---)	0.368	0.871	(---)
TF	Accepted	(---)	(---)	(---)	0.419	0.803	(---)
VE	Accepted	(---)	(---)	(---)	0.279	0.825	(---)
VF	Accepted	(---)	(---)	(---)	0.254	0.872	(-)
VG	Accepted	(---)	(---)	(---)	0.304	0.775	(---)
VI	Accepted	(---)	(---)	(---)	0.428	0.865	(-)
VK	Accepted	(--)	(---)	(---)	0.599	0.732	(---)
VL	Accepted	(---)	(---)	(---)	0.39	0.836	(--)
VN	Accepted	(---)	(---)	(---)	0.309	0.498	(---)
VQ	Accepted	(---)	(---)	(---)	0.332	0.506	(---)
VS	Accepted	(---)	(---)	(---)	0.674	0.817	(---)
VT	Accepted	(---)	(---)	(---)	0.559	0.893	(---)
YA	Accepted	(---)	(---)	(---)	0.277	0.894	(---)
YF	Accepted	(--)	(---)	(---)	0.181	0.921	(-)
YL	Accepted	(---)	(---)	(---)	0.299	0.909	(--)
YV	Accepted	(---)	(---)	(---)	0.294	0.91	(--)

HIA: Human Intestinal Absorption; $F_{20\%}$: Bioavailability 20%; $F_{30\%}$: Bioavailability 30%; VD: Volume Distribution, empirical decision 0.04-20: excellent (green), otherwise poor (red); T1/2: half-life; ROAT: Rat Oral Acute Toxicity. Empirical decision: Green-Excellent, Yellow-Medium, Red-Poor.

Figure S1. Docking validation of the crystallographic structures of Omarigliptin (PDB: 4PNZ) with the human DPP-IV.

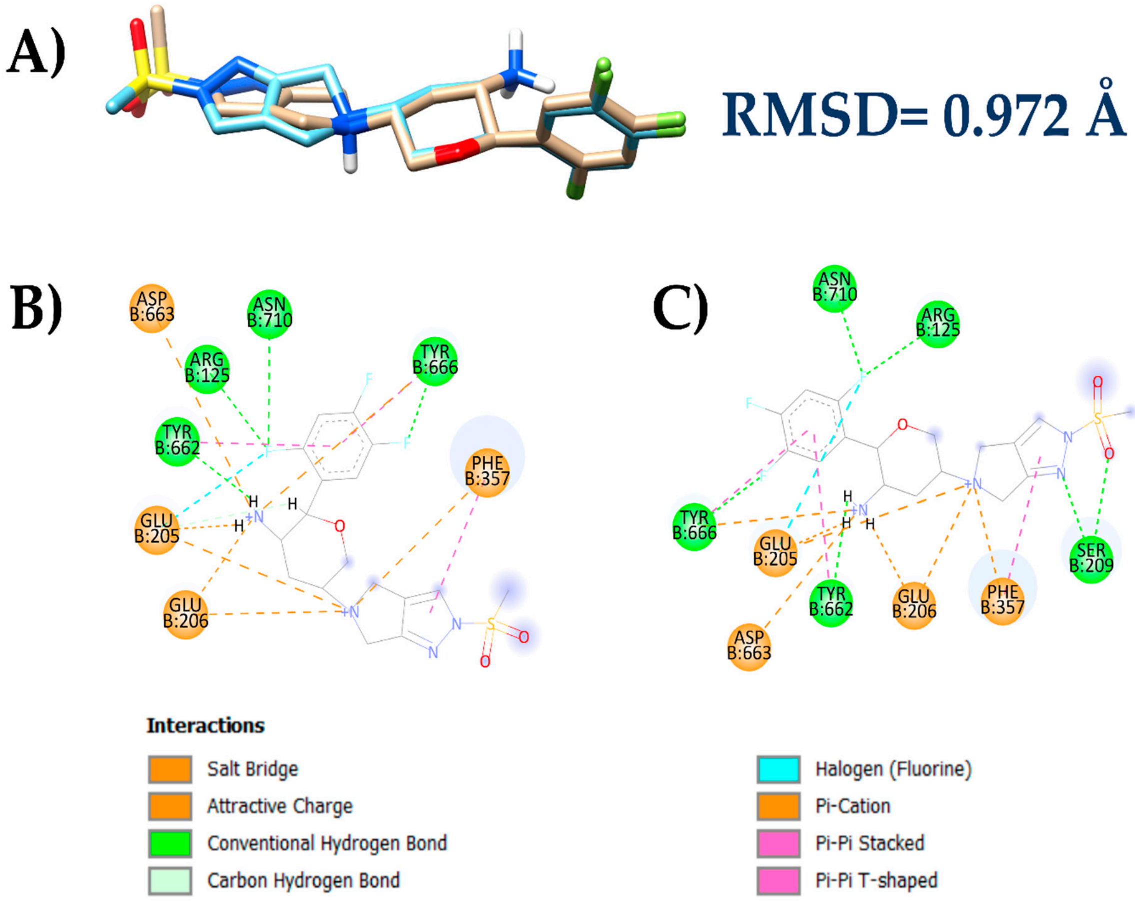

A) Overlap poses of the crystallographic Omarigliptin and the best-docked conformation of the Omarigliptin; B) Molecular interactions of the best-docked conformation of Omarigliptin with the active site of DPP-IV; and C) Molecular interactions of the crystallographic Omarigliptin with the active site of DPP-IV Acronyms. RMSD: Root median square deviation.
